# Anxiolytic-like effects of translocator protein (TSPO) ligand ZBD-2 in an animal model of chronic pain

**DOI:** 10.1186/s12990-015-0013-6

**Published:** 2015-03-26

**Authors:** Dong-sheng Wang, Zhen Tian, Yan-yan Guo, Hong-liang Guo, Wen-bo Kang, Shuo Li, Ya-ting Den, Xu-bo Li, Bing Feng, Dan Feng, Jian-ning Zhao, Gang Liu, Ming-gao Zhao

**Affiliations:** Department of Orthopedics, Jinling Hospital, Clinical School of Nanjing, Second Military Medical University, Nanjing, 210002 China; Department of Pharmacology, School of Pharmacy, Fourth Military Medical University, Xi’an, 710032 China

**Keywords:** Translocator protein (18 kDa), Anxiety, Pain, Amygdala

## Abstract

The activation of Translocator protein (18 kDa) (TSPO) has been demonstrated to mediate rapid anxiolytic efficacy in stress response and stress-related disorders. This protein is involved in the synthesis of endogenous neurosteroids that promote γ-aminobutyric acid (GABA)-mediated neurotransmission in the central neural system. However, little is known about the functions and the underlying mechanisms of TSPO in chronic pain-induced anxiety-like behaviors. The novel TSPO ligand *N*-benzyl-*N*-ethyl-2-(7,8-dihydro-7-benzyl-8-oxo-2-phenyl-9H-purin-9-yl) acetamide (ZBD-2) was used in the present study. We found that ZBD-2 (0.15 or 1.5 mg/kg) significantly attenuated anxiety-like behaviors in mice with chronic inflammatory pain induced by hindpaw injection of complete Freund’s adjuvant (CFA). However, the treatment did not alter the nociceptive threshold or inflammation in the hindpaw. Hindpaw injection of CFA induced the upregulation of TSPO, GluR1-containing α-amino-3-hydroxy-5-methyl-4-isoxazolepropionic acid (AMPA) receptors, and NR2B-containing N-methyl-d-aspartate (NMDA) receptors in the basolateral amygdala (BLA). ZBD-2 administration reversed the alterations of the abovementioned proteins in the BLA of the CFA-injected mice. Electrophysiological recording revealed that ZBD-2 could prevent an imbalance between excitatory and inhibitory transmissions in the BLA synapses of CFA-injected mice. Therefore, as the novel ligand of TSPO, ZBD-2 induced anxiolytic effects, but did not affect the nociceptive threshold of mice under chronic pain. The anxiolytic effects of ZBD-2 were related to the regulation of the balance between excitatory and inhibitory transmissions in the BLA.

## Introduction

The translocator protein (18 kD) (TSPO) is mainly located in the outer mitochondrial membrane in peripheral tissues and the central nervous system (CNS). This protein was initially identified as a peripheral binding site for diazepam and later functionally and structurally distinguished from the central benzodiazepine receptor [1]. It is mainly located in the outer mitochondrial membrane and favors the transport of cholesterol to the inner mitochondrial membrane, ultimately promoting neurosteroid synthesis [1]. TSPO is not directly targeted to the GABA_A_ receptor [2]. Ligands of TSPO such as XBD173, YL-IPA08, and AC-5216 exert anxiolytic and anti-depressant effects in animal models [3-5]. Its levels are modulated in anxiety disorders, such as in stress response [6] and mood disorders [1]. Upregulation of TSPO has been observed in response to injury and neurodegenerative diseases, such as multiple sclerosis, amyotrophic lateral sclerosis, Parkinson’s disease, Huntington’s disease, Alzheimer’s disease, and stroke [7]. However, little is known regarding the roles and the underlying mechanisms of TSPO in chronic pain-induced anxiety-like behaviors.

Pain is a multidimensional experience that includes sensory-discriminative, emotional-affective, and cognitive components. Chronic pain is usually correlated with allodynia and hyperalgesia [8]. Clinically, patients with chronic pain have a greater tendency to develop mild psychiatric disorders [9]. Chronic pain induces the development of anxiety and depression, which eventually reduces the quality of life [10-12]. Previous studies have shown that anxiety behavior could be induced by an injection of complete Freund’s adjuvant (CFA) into the hindpaw of mice with persistent inflammatory pain [12]. In rats with CFA-induced monoarthritis, a TSPO agonist retarded or prevented the development of mechanical allodynia and thermal hyperalgesia in a dose dependent manner, thereby suggesting that spinal TSPO is involved in the development and maintenance of inflammatory pain behaviors [13].

Glutamate is a major excitatory neurotransmitter in the CNS. The NR2A- and NR2B-containing *N*-methyl-d-aspartate (NMDA) receptors are supposedly linked to different intracellular cascades and have different roles in synaptic plasticity [14]. The amygdala is a key component of the CNS that coordinates negative emotional responses to threatening stimuli. This structure consists of several anatomically and functionally distinct nuclei, including the lateral (LA) and basolateral (BLA) nuclei as well as the central nucleus (CeA) [15,16]. Studies on amygdala function have focused on the plasticity at the sensory inputs from the thalamus and cortex to the LA and BLA [17,18]. In addition, a balance between excitatory and inhibitory transmission is critical for physiological anxiety; the prolonged disturbance of this balance can promote pathological anxiety-like behaviors [19,20]. Studies demonstrated that TSPO agonists have anxiolytic effects that are mediated by endogenous GABAergic neurosteroids [3,21,22]. However, the role of TSPO in modulating the balance between excitatory and inhibitory transmissions in the BLA remains unknown.

**Figure 1:**
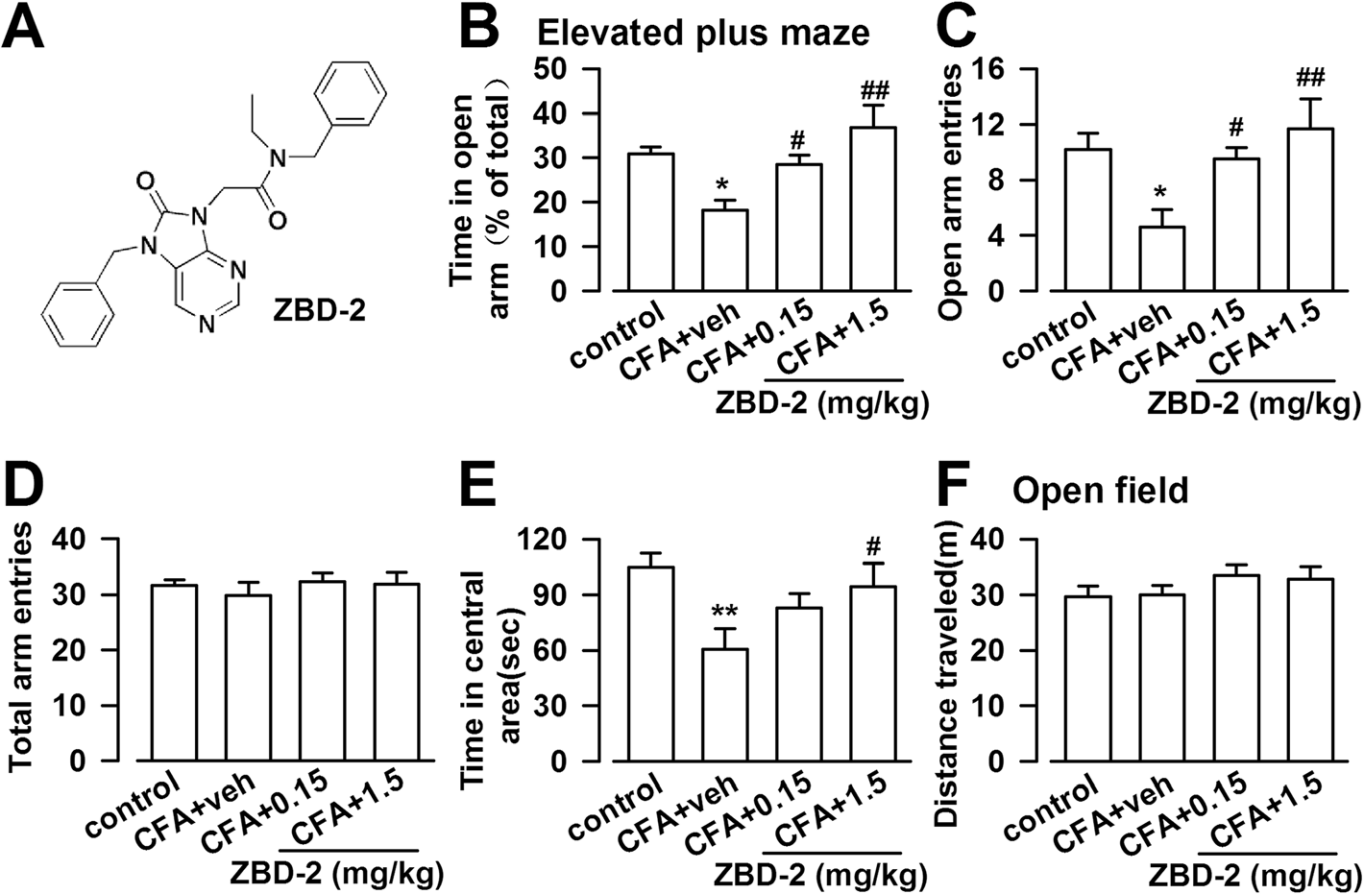
**ZBD-2 reduces anxiety-like behaviors. (A)** Chemical structures of ZBD-2. **(B)** EPM test were performed on Day 21 after CFA injection. ZBD-2 (0.15 and 1.5 mg/kg) reversed the time in the open arms in CFA-injected mice. **(C)** ZBD-2 (0.15 and 1.5 mg/kg) reversed the open arm entries in CFA-injected mice. **(D)** No difference of the total number of arm entries in each group. **(E)** In OF test, ZBD-2 (0.15 and 1.5 mg/kg) reversed the time in center areas in CFA-injected mice. **(F)** No difference of the total distance traveled in each group. n = 6 in each group, *p < 0.05, **p < 0.01 compared to the control group; ^#^ *p* < 0.05, ^##^ *p* < 0.01 compared to the CFA+ saline group.

The present study aimed to investigate the possible anxiolytic effects of a novel TSPO ligand N-benzyl-N-ethyl-2-(7,8-dihydro-7-benzyl-8-oxo-2-phenyl-9H-purin-9-yl) acetamide (ZBD-2; Figure 1A) and to elucidate the underlying mechanisms. ZBD-2 is a novel ligand for TSPO synthesized in our laboratory (Chinese patent number 201210047188.6). ZBD-2 is an analog of the XBD173 [3], one of the ligand of TSPO. ZBD-2 showed high affinity for TSPO prepared from rat brain mitochondria in the nanomolar range (Ki = 0.463 nM), comparable to the affinity of ^3^H-PK11195, the classic TSPO ligand, for TSPO (Ki = 0.657 nM) [5]. Here, we report that the activation of TSPO by ZBD-2 attenuates chronic pain-induced anxiety-like behaviors by regulating the balance between GABAergic and glutamatergic transmission in the BLA of hindpaw CFA-injected mice.

## Results

### Anxiolytic effects of ZBD-2 in mice with CFA-induced chronic inflammatory pain

Hindpaw CFA injection induced significantly anxiogenic behaviors that were detected at 21 d post-injection. In the EPM test, mice spent less time in the open arms (F _(3, 20)_ = 7.065, *p* = 0.002, LSD test; Figure 1B), and the number of open arm entries decreased (F _(3, 20)_ = 4.473, *p* = 0.015, LSD test; Figure 1C). The time in the central area was also decreased in the open field (OF) tests (F _(3, 20)_ = 3.902, *p* = 0.024, LSD test; Figure 1E). However, the total number of arm entries in the EPM tests and the total distance traveled in the OF tests did not change significantly, as compared with the controls (Figure 1D and F). This observation shows that locomotor activity was not significantly different between the groups. Administration of ZBD-2 (0.15 and 1.5 mg/kg) for one week reversed the anxiety-like behaviors in a dose-dependent manner as shown by the time spent in the open arm and the number of open arm entries in the EPM tests (Figure 1B and C) as well as the time spent in the central area in OF tests (Figure 1E).

### Effect of ZBD-2 on pain perception and inflammation

**Figure 2:**
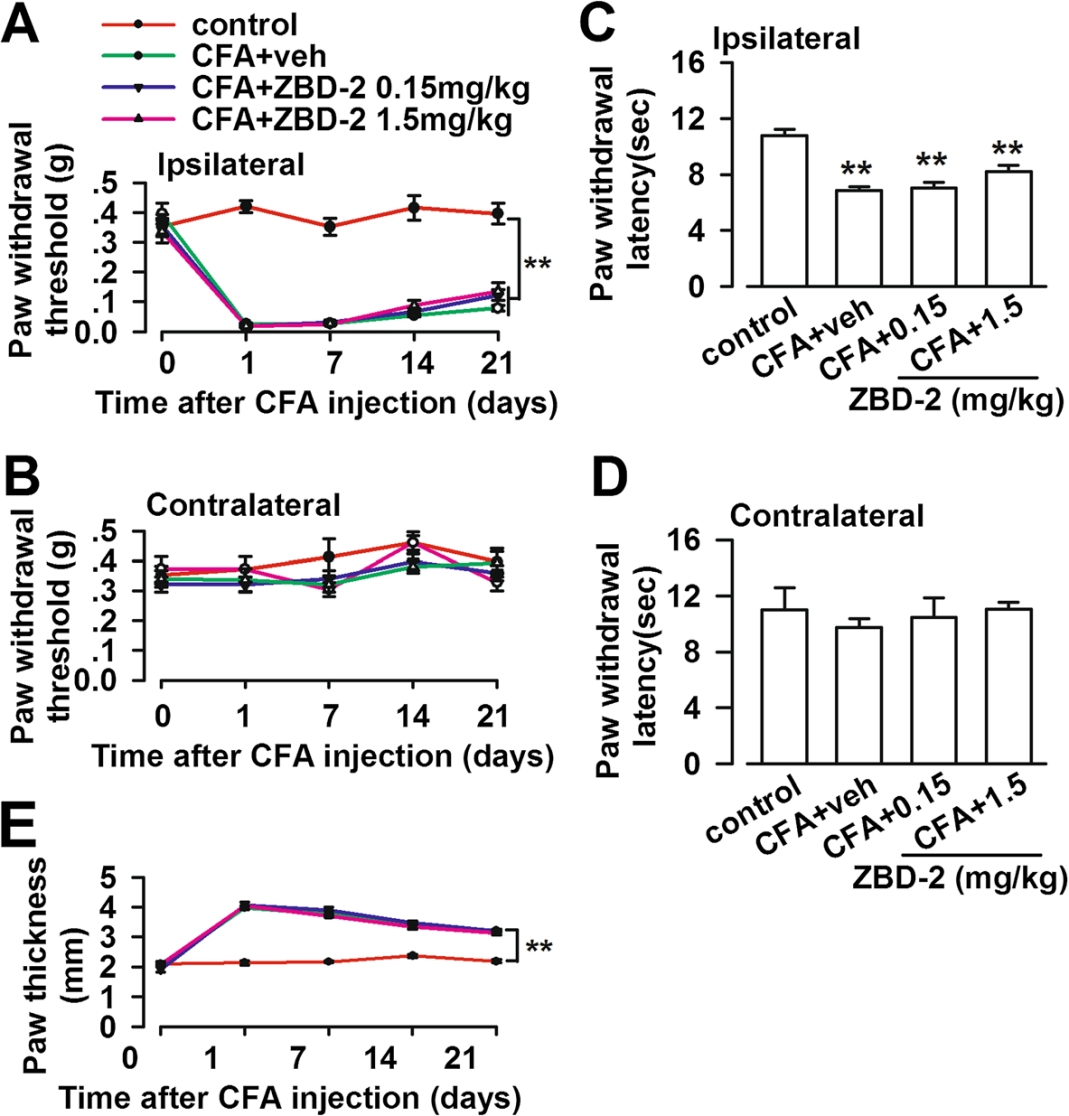
**Effects of ZBD-2 on pain perception.** Mechanical allodynia and thermal hyperalgesia were detected on Day 0, 1, 7, 14 and 21 after CFA injection. ZBD-2 (0.15 and 1.5 mg/kg) did not change the mechanical allodynia in the ipsilateral **(A)** and contralateral hindpaw **(B)**. ZBD-2 (0.15 and 1.5 mg/kg) did not change the thermal hyperalgesia in the ipsilateral **(C)** and contralateral hindpaw **(D). (E)** ZBD-2 did not reduce edema in CFA-injected hindpaws. n = 6 in each group, **p < 0.01 compared to the control group.

Subsequently, we determined whether the anxiolytic effects of ZBD-2 are caused by analgesic activity in animals with chronic pain. Tactile allodynia and thermal hyperalgesia were examined during the experimental period. However, we found that anxiolytic doses of ZBD-2 (0.15 and 1.5 mg/kg) did not reduce mechanical allodynia (Figure 2A) and thermal hyperalgesia (Figure 2C). The paw withdrawal threshold or latency was not significantly changed in the contralateral hindpaw (Figure 2B and D). Furthermore, ZBD-2 did not reduce chronic inflammation, as shown by the unchanged edema in CFA-injected hindpaws (Figure 2E). These results suggested that the anxiolytic effects of ZBD-2 were not caused by anti-inflammatory or analgesic activities.

### Reversed up-regulation of TSPO by ZBD-2 in the amygdala

**Figure 3:**
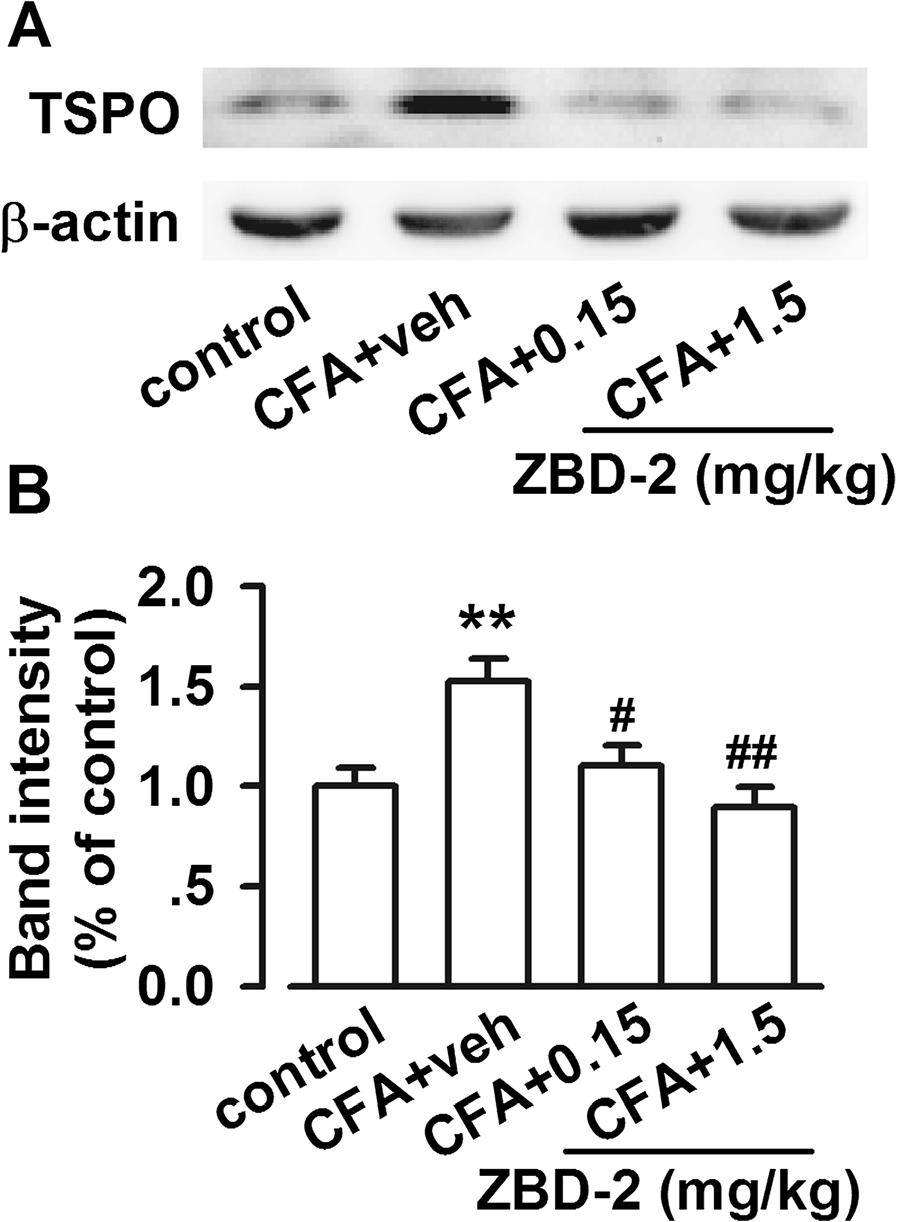
**Effects of ZBD-2 on TSPO levels. (A)** Representative results of Western blot analysis for TSPO in the BLA on the Day 21 after hindpaw CFA-injection. **(B)** ZBD-2 (0.15 and 1.5 mg/kg) for one week significantly decreased the upregulation of TSPO in the BLA. n = 6 in each group, **p < 0.01 compared to the control group; ^#^ *p* < 0.05, ^##^ *p* < 0.01 compared to the CFA+ saline group.

Increased TSPO expression is observed in neurological disorders, such as traumatic brain injury and inflammation [23]. Levels of TSPO in the BLA were increased on day 21 after hindpaw CFA injection (F _(3, 20)_ = 7.081, *p* = 0.002, LSD test; Figure 3). Treatment of ZBD-2 (0.15 and 1.5 mg/kg) for one week significantly decreased the upregulation of TSPO in the BLA (Figure 3). These results indicated that TSPO expression in the BLA is a response to chronic painful stimuli.

### Effects of ZBD-2 on synaptic proteins expression in the BLA

**Figure 4:**
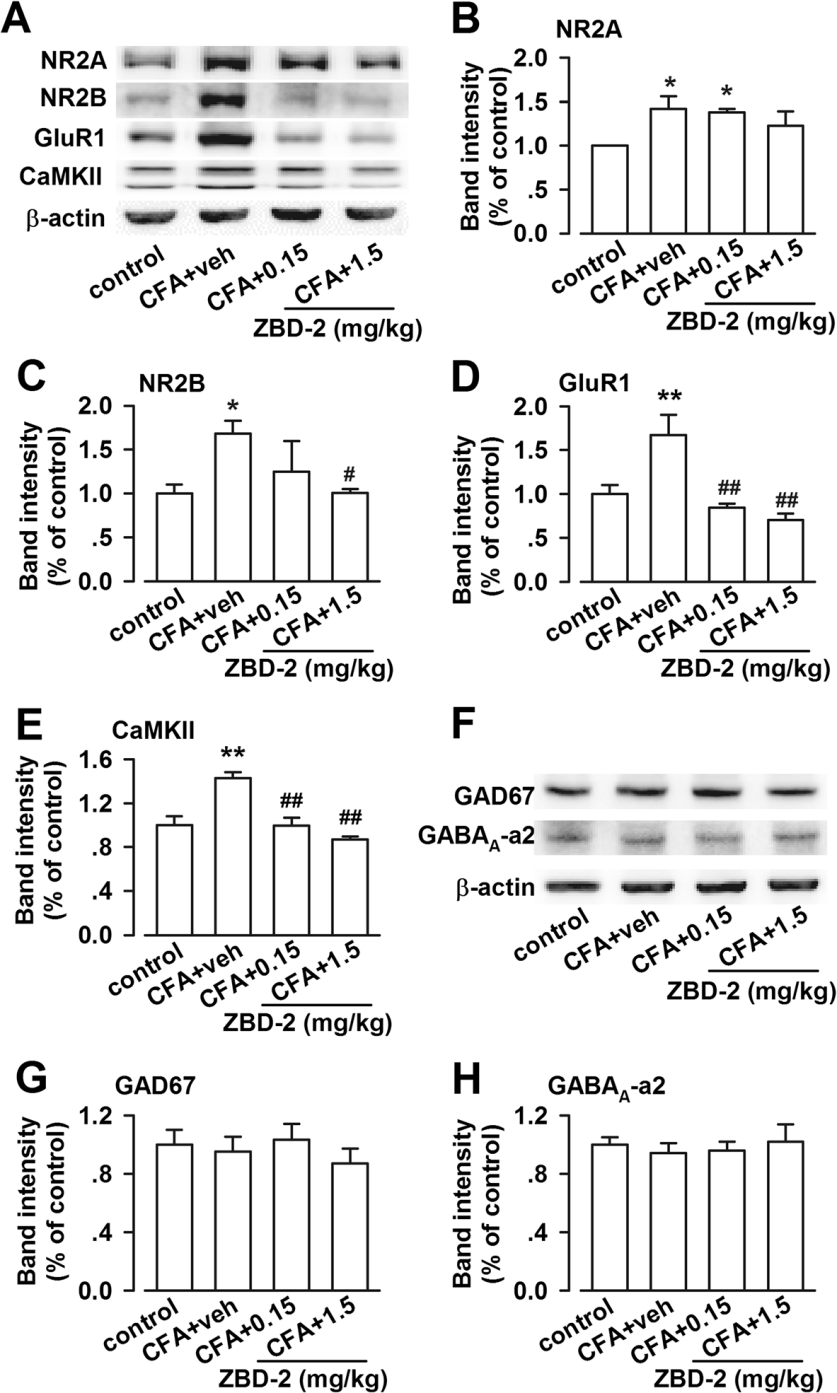
**Effects of ZBD-2 on protein expression in the BLA. (A)** Representative results of Western blot analysis in the BLA on the Day 21 after hindpaw CFA-injection. **(B)** ZBD-2 (0.15 and 1.5 mg/kg) for one week slightly decreased the levels of NR2A-containing NMDARs. **(C)** ZBD-2 (1.5 mg/kg) reversed the upregulation of NR2B-containing NMDARs. **(D-E)** ZBD-2 (0.15 and 1.5 mg/kg) reversed the upregulation of GluR1 and CaMKII-α. **(F)** Representative results of Western blot analysis for GABA_A_-α2 and GAD67. **(G and H)** Levels of GABA_A_-α2 and GAD67 were not altered in groups. n = 6 in each group, *p < 0.05, **p < 0.01 compared to the control group; ^#^ *p* < 0.05, ^##^ *p* < 0.01 compared to the CFA+ saline group.

An imbalance between the excitatory and inhibitory transmission in the amygdala promotes emotional disorders [20]. First, we detected the levels of proteins related to the excitatory synaptic transmission (Figure 4A). The levels of NR2A- and NR2B-containing NMDARs (F _(3, 20)_ = 3.118, *p* = 0.049, LSD test, Figure 4B; F _(3, 20)_ = 3.313, *p* = 0.041, LSD test, Figure 4C) as well as GluR1 (F _(3, 20)_ = 10.864, *p* = 0.000, LSD test; Figure 4D) were remarkably increased after CFA-injection. The critical excitatory synaptic protein Ca^2+^ /calmodulin-dependent protein kinase II-α (CaMKII-α) also significantly increased after CFA injection (F _(3, 20)_ = 16.874, *p* = 0.000, LSD test; Figure 4E). Treatment with ZBD-2 (0.15 and 1.5 mg/kg) for one week significantly reversed the up-regulation of the abovementioned proteins in the BLA (Figure 4A–E). However, no significant differences were observed in the levels of GABA_A_-α2 and GAD67, which is a glutamate decarboxylase that is critical for GABA synthesis (Figure 4F-H).

### Effects of ZBD-2 on excitatory synaptic transmissions in the BLA

**Figure 5:**
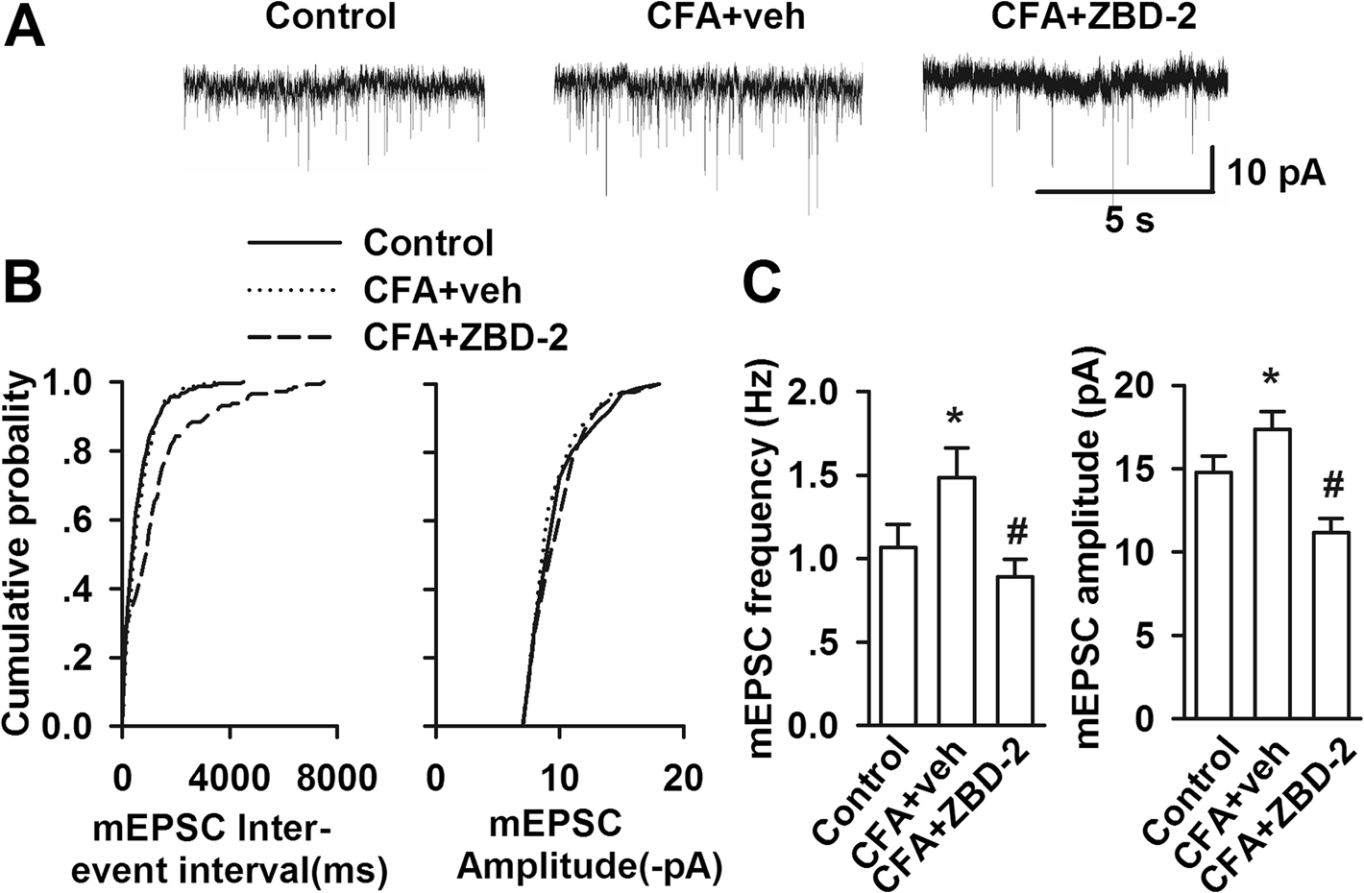
**Effects of ZBD-2 on mEPSC in the BLA. (A)** Representative mEPSCs recorded in pyramidal neurons of BLA at a holding potential of-70 mV. **(B)** Cumulative frequency and amplitude histogram of the mEPSCs from the slices in each group. **(C)** Summery of mEPSCs frequency (left) and amplitude (right) in control (n = 11 slices/4 mice), CFA-vehicle (n = 10 slices/4 mice), CFA-ZBD-2 (n = 12 slices/4 mice) treated mice. ZBD-2 (1.5 mg/kg) for one week reversed the increases of mEPSCs frequency and amplitude in CFA-treated mice. *p < 0.05 compared to the control group; ^#^ *p* < 0.05 compared to the CFA+ saline group.

To further determine the role of TSPO in the excitatory synaptic transmissions in the BLA, we performed whole-cell patch-clamp recordings and recorded miniature excitatory postsynaptic currents (mEPSC) in the pyramidal neurons of BLA after three weeks since the CFA injection (Figure 5A). The frequency and the amplitude of mEPSC were significantly increased in the BLA neurons of CFA-injected mice (F _(2, 15)_ = 5.285, *p* = 0.018, LSD test, Figure 5B; F _(2, 15)_ = 10.900, *p* = 0.001, LSD test, 5C). ZBD-2 (1.5 mg/kg) could reverse the enhancement of mEPSCs induced by CFA injection (Figure 5).

### Effects of ZBD-2 on inhibitory synaptic transmissions in the BLA

**Figure 6:**
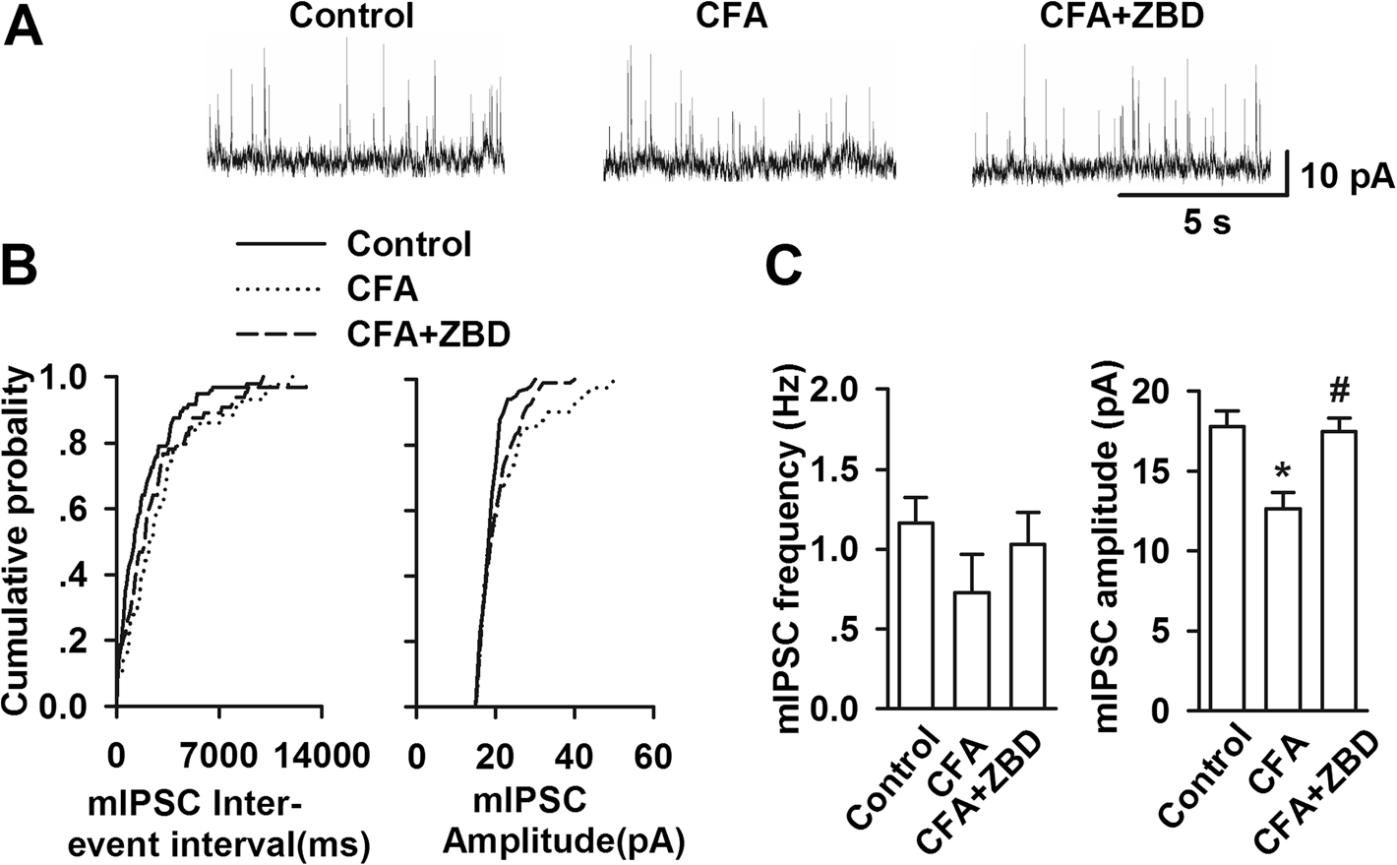
**Effects of ZBD-2 on mIPSC in the BLA. (A)** Representative mIPSCs recorded in pyramidal neurons of BLA at a holding potential of 0 mV. **(B)** Cumulative frequency and amplitude histogram of the mIPSCs from the slices in each group. **(C)** Summery of mIPSCs frequency (left) and amplitude (right) in control (n = 12 slices/4 mice), CFA-vehicle (n = 12 slices/4 mice), CFA-ZBD-2 (n = 11 slices/4 mice) treated mice. ZBD-2 (1.5 mg/kg) for one week reversed the decreases of mIPSCs amplitude in CFA-treated mice. *p < 0.05 compared to the control group; ^#^ *p* < 0.05 compared to the CFA+ saline group.

The GABA_A_ receptor-mediated miniature inhibitory postsynaptic currents (mIPSC) were recorded in BLA pyramidal neurons after three weeks of CFA-injection (Figure 6A and B). The mIPSC frequency could not detect significant differences among the mice from different treatment groups. However, the mIPSC amplitude was significantly decreased in CFA-injected mice (F _(2, 15)_ = 8.856, *p* = 0.003, LSD test; 6C). Treatment with ZBD-2 (1.5 mg/kg) recovered the mIPSC amplitude alteration that was induced by CFA injection (Figure 6C).

## Discussion

In the present study, pain caused by chronic inflammation was found to induce pronounced anxiety-like behaviors. Activation of TPSO with ZBD-2 induced notable anxiolytic effects in mice with chronic pain, but did not alter the nociceptive threshold and the inflammation. The anxiolytic effects of ZBD-2 were related to the regulation of synaptic transmission in the BLA.

Patients coping with inflammatory arthritis commonly develop anxiety [24]. The pathophysiological features and duration of CFA-induced pain in model organisms make the study of pain and pain associated behaviors possible [12]. In the hindpaw CFA-injected mouse model, chronic pain behaviors were verified by hyperalgesia and allodynia, whereas the anxiety-like behaviors were assessed with EPM and OF, which evaluate exploratory behaviors associated with anxiety. Administration of ZBD-2 (0.15 and 1.5 mg/kg) for one week attenuated anxiety-like behavior in a dose-dependent manner without affecting the locomotor activity. The anxiolytic dose of ZBD-2 (0.15 and 1.5 mg/kg) was similar to XBD173 (0.1, 1, 10 mg/kg in rat) and A C-5216 (the light/dark box test in rats 0.1–3 mg/kg and, social interaction test in mice 0.01–0.3 mg kg) [5]. However, the LD50 of ZBD-2 was over 3 g/kg (unpublished data).

The expression of TSPO increased in mice with chronic pain. In the healthy nervous system, TSPO is expressed at low levels in glia, as well as in some populations of neurons [25]. By contrast, TSPO is highly expressed in the injured or diseased central or peripheral nervous system at the lesion sites. In the CNS, TSPO is up-regulated in microglia, astrocytes, and infiltrating macrophages, as well as occasionally in neurons [7]. This upregulation may be the compensatory effects in response to injury and neurodegenerative diseases. Down-regulation of TSPO by ligands of TSPO may result from the attenuation of the compensatory effects. Activation of TSPO is beneficial for several kinds of neurological diseases by promoting neurosteroid synthesis [25]. Such diseases include injury to peripheral nerves or the brain [26], multiple sclerosis [27], Alzheimer’s disease [28], and post-traumatic stress disorder [29,30]. The TSPO agonist Ro5-4864 diminishes mechanical allodynia and thermal hyperalgesia induced by CFA-induced monoarthritis [13] and prevents the first- and second-phase responses in the formalin test [31]. These data suggest that activation of TSPO is effective for treatment of inflammatory pain [32]. However, our findings in the present study were not consistent with these studies. In the hindpaw CFA injection models, we found that the activation of TSPO with ZBD-2 did not diminish the mechanical allodynia and thermal hyperalgesia; the chronic inflammation ware also not reduced in CFA-injected hindpaws. The discrepancy may be attributed to the use of different pain models. Hernstadt et al. [13] showed that TSPO ligands diminished mechanical allodynia and thermal hyperalgesia in the CFA-induced monoarthritis model. Present results with the hindpaw CFA injection induced long-term inflammation in the paw, as shown by the unchanged edema. Detections of mechanical allodynia and thermal hyperalgesia were applied on the plantar surface of left hindpaw that was the direct site of CFA injection. However, the site of CFA injection was tibiotarsal joint in monoarthritis model. Furthermore, the present data provided the evidences that ZBD-2 could regulated the synaptic transmission in the BLA. These results suggested that the anxiolytic effects of ZBD-2 were not caused by anti-inflammatory or analgesic activity.

The amygdala is a critical region involved in the integration of pain and anxiety [33,34]. The GABA system is well-known to have a major role in the pathogenesis of anxiety and fear conditioning [35]. The improved excitability of output neurons in the basolateral amygdala improves aversive conditioning, whereas decreasing the excitability of these neurons produces anxiolytic effects [36]. Our previous study reported that levels of excitatory glutamate receptors increased, whereas the levels of inhibitory GABA receptors decreased in the stressed mice with anxiety behavior [20]. In the present study, we found that the activation of TSPO by ZBD-2 enhanced GABAergic neurotransmission, whereas ZBD-2 did not affect the levels of GABA_A_-α2 and GAD67, which is critical for GABA synthesis. This result is consistent with the findings that TSPO activation indirectly enhances the GABAergic neurotransmission via the generation of GABAergic neurosteroids [3]. In addition, administration of ZBD-2 reversed the up-regulation of excitatory glutamate receptors, including the NR2A- and NR2B-containing NMDARs, GluR1 receptors, and the excitatory synaptic protein CaMKII in the BLA from CFA-injected mice. CaMKII-mediated phosphorylation acutely regulates the function and trafficking of postsynaptic substrates in response to synaptic activity [37]. High levels of CaMKII which is response to chronic pain implies the enhanced excitatory synaptic transmission. Electrophysiological recording provided further evidence that activation of TSPO with ZBD-2 maintained the balance between excitatory and inhibitory synaptic transmission in neural circuits of the amygdala.

In conclusion, the present data provides strong evidence for the role of ZBD-2 in reducing anxiety-like behavior in animals with persistent inflammatory pain. ZBD-2 may play a role in the regulation of pain and anxiety through endogenous neurosteroid production, which modulates cellular and receptors components. The underlying mechanisms involved the regulation of neuronal circuits in the basolateral amygdala.

## Materials and methods

### Materials

All chemicals were obtained from Sigma (St. Louis, MO) unless otherwise stated. ZBD-2 was synthesized at our Laboratory with the purity of 99.9% and dissolved in the saline (0.9% NaCl). Rabbit anti-NR2A, mouse anti-NR2B, and mouse anti-GAD67 antibodies were purchased from Millipore (Billerica, MA). Mouse anti-CaMKIIα antibody was purchased from Santa Cruz Biotech (Santa Cruz, CA, USA). Rabbit anti-GluR1 antibody was purchased from Abcam (Cambridge, UK). All of the chemicals and reagents used were commercially available and of standard biochemical quality.

### Animals

Adult (8 weeks old) male C57BL/6 mice from the Fourth Military Medical University Experimental Animal Center were used for the experiments. The animals were housed in groups with a 12 h light: 12 h dark cycle (light on 7:00 AM) at room temperature (24 ± 2°C), humidity (50%–60%), the food and water were allowed freely. Animals were allowed to accommodate to laboratory conditions for 7 days before the procedure. All behavioral testing occurred during the light period on the designated day of experiment. All experimental procedures were approved by the Animal Ethics Committee of the Fourth Military Medical University. To induced inflammatory pain, mice were injected subcutaneously with CFA (10 μl, 50% in saline) into the plantar surface of left hindpaw. The control mice were injected with the same volume saline. The paw volume was assessed using a Vernier caliper to determine the diameter of edema. Two weeks after CFA administration, the animals received an orally administration of vehicle or ZBD-2 (0.15 mg/kg or 1.5 mg/kg) once a day for 6 days, i.e., from Day 16 to Day 21 after CFA injection. Behavioral tests were performed during the experiments. Brain samples for western blot were dissected immediately after behavior tests on Day 21.

### Thermal hyperalgesia

Thermal hyperalgesia was assessed by measuring the latency of paw withdrawal in response to a radiant heat source [38]. To assess thermal nociceptive responses, a commercially available plantar analgesia instrument (BME410A, Institute of Biological Medicine, Academy of Medical Science, China) was used. Animals were placed in individual plastic boxes and allowed to adjust to the environment for 25 min. Thermal hyperalgesia was assessed by measuring the paw withdrawal latency (PWL) in response to a radiant heat source. The heat source was turned off when the mice lifted the foot, allowing the measurement of time from onset of radiant heat application to withdrawal of the mice’s hindpaw. This time was defined as the PWL. Left paws were tested at 5 minutes intervals for a total of five trials. A 20 s cutoff was used to prevent tissue damage.

### Mechanical allodynia

Mice were placed in individual plastic boxes and allowed to adjust to the environment for 25 min. Using the up-down paradigm [38], mechanical sensitivity was assessed with a set of von Frey filaments. Based on preliminary experiments that characterized the threshold stimulus in untreated animals, the innocuous 0.4 mN (#2.44) filament, representing 50% of the threshold force, was used to detect mechanical allodynia. The filament was applied to the point of bending six times each to the dorsal surface of the hindpaw. Positive responses included prolonged hindpaw withdrawal followed by licking or scratching. For each time point, the percentage response frequency of hindpaw withdrawal was expressed as follows: (number of positive responses)/6 × 100 per hindpaw.

### Elevated plus maze (EPM)

The Elevated Plus Maze (EPM) was conducted as described in a previous report [39]. The apparatus comprised two open arms (25 × 8 × 0.5 cm) and two closed arms (25 × 8 × 12 cm) that extended from a common central platform (8 × 8 cm). The apparatus was elevated to a height of 50 cm above the floor. Mice were allowed to habituate to the testing room for 2 days before the test, and pretreated with gentle handling two times per day to eliminate nervousness. For each test, individual animals were placed in the center square, facing an open arm, and allowed to move freely for 5 min. Mice were videotaped using a camera fixed above the maze and analyzed with a video-tracking system. The number of entries and time spent in each arm were recorded.

### Open field test

The open field (OF) test was conducted as described in a previous report [39]. The apparatus (JL Behv-LAM-Shanghai jiliang software, china) was a square arena (30 × 30 × 30 cm) with clear Plexiglas walls and white floor and was placed inside an isolation chamber with dim illumination. Mice were placed in the center of the box and allowed to adjust to the environment for 10 minutes. Mice were videotaped using a camera fixed above the floor and analyzed with a video-tracking system. The “center” field is defined as the central 15 × 15 cm^2^ area of the open field, one-fourth of the total area. Each subject was placed in the center of the open field, and its activity was measured for 15 min.

### Western blot

Western blot analysis was performed as described previously [39]. Tissue samples from the amygdala were dissected from the brain slices under the anatomical microscope. Equal amounts of protein (50 μg) were separated and electro transferred onto PDVF membranes (Invitrogen), which were probed with antibody for TSPO (dilution ratio 1:2000), NR2A (dilution ratio 1:200), and NR2B (dilution ratio 1:500), GAD67 (dilution ratio 1:1500), CaMKII-α (dilution ratio 1:2000) and GluR1 (dilution ratio 1:300) with β-actin (dilution ratio 1:10000) as the loading control. The membranes were incubated with horseradish peroxidase–conjugated secondary antibodies (anti-rabbit/anti-mouse/anti-goat IgG for the primary antibodies). The densitometric analysis of the Western-blot was conducted using a ChemiDoc XRS (Bio-Rad, Hercules, CA) and quantified using Quantity One version 4.1.0 (Bio-Rad) according to the instructions. For data quantification, band intensity of each blot was calculated as ratio relative to β-actin. The intensity ratio of the control group was set as 100%, and the intensity of other treatment groups were expressed as percentage to the control group.

### Whole-cell patch-clamp recording

Mice were anesthetized with 4% isoflurane in air and then decapitated. The brains were rapidly removed and placed into ice cold artificial cerebrospinal fluid (ACSF). Transverse slices (300 μm) containing the BLA were cut and transferred to a recovery chamber, and was submerged in oxygenated (95% O_2_ and 5% CO_2_) ACSF at room temperature. After 1 h of recovery, slices were placed in a recording chamber on the stage of an Olympus microscope with infrared digital interference contrast optics for visualization of whole-cell patch-clamp recordings with an Axopatch 200B amplifier (Axon Instruments, Union City, CA, USA). Recordings were performed at room temperature (21°C–23°C), with continuous perfusion of ACSF at a rate of 2 ml/min. Miniature EPSCs (mEPSCs) were recorded from BLA pyramid neurons clamped at-70 mV with the picrotoxin (20 μM) in the ACSF. The recording pipettes (3–5 MΩ) were filled with solution containing the following (in mM): 145K-gluconate, 5 NaCl, 1 MgCl_2_, 0.2 EGTA, 10 HEPES, 2 Mg-ATP, and 0.1 Na3-GTP, adjusted to pH 7.2 with KOH. TTX (0.5 μM) was added in the perfusion solution. Miniature inhibitory postsynaptic currents (mIPSCs) were collected at 0 mV with the CNQX (20 μM) and AP-5 (50 μM) in the ACSF. The intracellular solution for mIPSCs was as follows: 102 mM cesium gluconate, 5 mM TEA-Cl, 3.7 mM NaCl, 10 mM BAPTA, 0.2 mM EGTA, 20 mM HEPES, 2 mM Mg-ATP, 0.3 mM Na_3_GTP, and 2.5 mM QX-314-Br, adjusted to pH 7.2 with CsOH (280–300 mOsmol). Access resistance (15–30 MΩ) was monitored throughout the experiment. Data were discarded if access resistance changed >15% during an experiment.

### Date analyses

Data were presented as the mean and standard errors of the means (SEM). Statistical analysis of differences between two groups was performed by independent sample, two-tailed *T* test. Data of multiple groups were evaluated using one-way analysis of variance (ANOVA) for post hoc comparisons (SPSS 13.0). Data that passed the homogeneity test were analyzed by the one-way ANOVA least significant difference (LSD) test. Data that did not pass the homogeneity test were analyzed by the one-way ANOVA Dennett’s T3 test. In all cases, *p <* 0.05 was considered statistically significant.

## Abbreviations

BLA: Basolateral amygdala
ACSF: Artificial cerebrospinal fluid
AMPAR: α-amino-3-hydroxy-5-methyl-4-isoxazolepropionic acid receptor
CFA: Complete Freund’s adjuvant
mEPSC: Miniature excitatory postsynaptic current
mIPSC: Miniature inhibitory postsynaptic current
GABA: γ-aminobutyric acid
NMDA: N-methyl-D-aspartic acid
TSPO: Translocator protein (18 kDa)
